# Active Electro-Location of Objects in the Underwater Environment Based on the Mixed Polarization Multiple Signal Classification Algorithm

**DOI:** 10.3390/s18020554

**Published:** 2018-02-11

**Authors:** Yidong Xu, Wenjing Shang, Lili Guo, Junwei Qi, Yingsong Li, Wei Xue

**Affiliations:** 1 College of Information and Communication Engineering, Harbin Engineering University, Harbin 150001, China; xuyidong@hrbeu.edu.cn (Y.X.); shangwenjing@hrbeu.edu.cn (W.S.); guolili@hrbeu.edu.cn (L.G.); xuewei@hrbeu.edu.cn (W.X.); 2 National Space Science Center, Chinese Academy of Sciences, Beijing 100190, China; 3 Acoustic Science and Technology Laboratory, Harbin Engineering University, Harbin 150001, China

**Keywords:** active electro-location, underwater, MP-MUSIC algorithm, electric dipole source, UCA

## Abstract

This article proposes a novel active localization method based on the mixed polarization multiple signal classification (MP-MUSIC) algorithm for positioning a metal target or an insulator target in the underwater environment by using a uniform circular antenna (UCA). The boundary element method (BEM) is introduced to analyze the boundary of the target by use of a matrix equation. In this method, an electric dipole source as a part of the locating system is set perpendicularly to the plane of the UCA. As a result, the UCA can only receive the induction field of the target. The potential of each electrode of the UCA is used as spatial-temporal localization data, and it does not need to obtain the field component in each direction compared with the conventional fields-based localization method, which can be easily implemented in practical engineering applications. A simulation model and a physical experiment are constructed. The simulation and the experiment results provide accurate positioning performance, with the help of verifying the effectiveness of the proposed localization method in underwater target locating.

## 1. Introduction

Detecting and estimating the target position underwater have many important applications including underwater localization, deep sea exploration and rescue missions in catastrophic conditions. However, underwater localization still remains a challenge in robotics [1,2,3]. In the last few decades, the acoustic-based locating method has played a main role in underwater localization [4,5,6]. The echolocation obtained by sonar is problematic because the varying temperature and density, Doppler effect and background noise might cause interferences to the signal [7,8]. The light- or map-based underwater localization method is restricted by the transparency of water, which cannot work in a dark or turbid environment [9]. To overcome these drawbacks, in fact, nature has already discovered an original sense to adapt well to this situation: the electric sense [1]. The electric fish has an electric organ of discharge located at the base of its tail, which can help it detect and locate the target in a dark and turbid environment. The electric organ of discharge generates a dipolar-shaped electric field around the fish, which can be distorted by the surrounding objects. Then, the fish “measures” the distortions of the electric field by using the electro-receptors distributed along its body and uses its brain to get an image of its surroundings [10,11]. This means that understanding and imitating the electric sense with technology would offer the opportunity to enhance the target detecting and locating abilities of underwater robots.

In this perspective, in [12], the authors set up an experiment by using four-point electrodes that are placed at the apexes of a rhombus in a rigid moving frame driven by a robot. In this system, two electrodes that are situated at the opposite apexes of the lozenge are polarized in voltage and play the role of the electric organ of discharge, while the other two opposite electrodes play the role of receivers. Rasnow [13] first applied a small sphere perturbation formula in a uniform electric field, which is still very popular in the latest electro-location literature [10,14,15,16]. However, in this theoretical model, the field near the target should be a uniform electric field, which means that the radius of spherical targets should be small. Thus, the surrounding field performs as the uniform electric field. In the works [17], an artificial electrosensory array was designed to study the feasibility in underwater robots. The artificial electrosensory array was able to estimate the position of a plastic sphere with a diameter of 10 mm, when the plastic sphere was 12 mm away from the electrosensory array. Their distance estimation algorithm was realized based on the spatial distribution of the sensor measurements [18]. In Lebastard’s work [3,19], a bio-inspired method has been proposed to improve the localization performance on the basis of the unscented Kalman filter. In Peng’s work [20], a finite element model (FEM) of underwater an active electrolocation system based on the coupling Cole–Cole model and Maxwell theory was built. However, in both Lebastard’s and Peng’s electro-location systems, the electrosensory array needs to move and measure the electric field while locating the target, which is not suitable for positioning a dangerous target in practice. In the work [21], a MUSIC-type algorithm is proposed for locating small inclusions buried in a half-space by means of measuring the scattering amplitude at a fixed frequency in two-dimensional space. The locating method is based on far area theory. However, far area theory is not suitable for underwater target locating because the high frequency radiation wave cannot be transferred a long distance.

Considering the challenges of the underwater target localization, this paper proposes a novel solution for underwater locating based on the boundary element method (BEM) theory and mixed polarization multiple signal classification (MP-MUSIC) algorithm. In this method, we first use the BEM to accurately describe the induction field of the metallic target and insulator target with Poisson’s equation. Then, a UCA system is designed, which acts as the underwater target locating sensors. In these locating sensors, two electrodes act as the dipole source, while five electrodes situated at the equipotential points of the dipole source play the role of receivers. One of the five electrodes is set as the reference point, and hence, we can measure the voltage between the other four electrodes and the reference point. Since the five electrodes of the UCA system are situated at the equipotential points of the dipole source, they cannot receive the primary field of the dipole source. However, they can receive the induction field from the target. In the locating procedure, we introduce the mixed polarization MUSIC algorithm, which provides good performance and does not suffer from the problem of non-convexity [22,23]. Different from the other MUSIC algorithms for radar, such as root-MUSIC and beamspace MUSIC, MP-MUSIC could deal with signal polarization, which is suitable for underwater electro-location. The position of the target can be located via finding the minimum eigenvalue of the estimated gain matrix and the project matrix of the noise subspace by using the MP-MUSIC algorithm [24]. We also propose a simplified locating model and introduce the Rasnow model and canonical MUSIC for comparison in this paper. The effectiveness of the proposed method is investigated and compared with the numerical model and simulation model. We also setup a physical experiment to verify the proposed locating method. The results show that the proposed algorithm is effective for underwater target locating.

## 2. Underwater Target Electro-Locating Method

### 2.1. Underwater Target Electro-Locating Model

**Figure 1:**
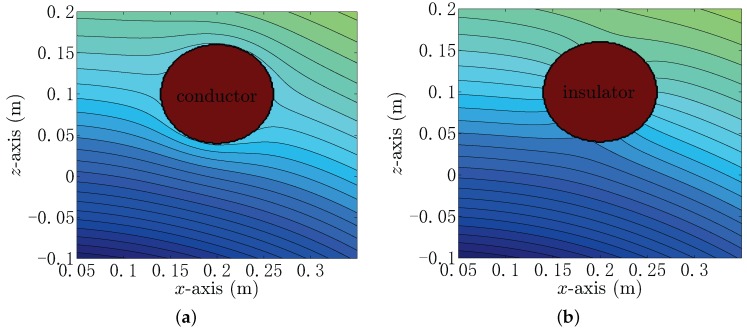
(**a**) An example of the potential distribution distorted by the spherical metallic target; (**b**) an example of the potential distribution distorted by the spherical insulator target.

To locate the target in the underwater environment, the electric field distribution should first be investigated. The field distribution can be distorted by a metallic or insulator target in the underwater environment, which is shown in Figure 1. Given the information of a target and the electric excitation source, the distortion can be evaluated by means of solving the forward problem model. The location of the target can be estimated by the use of the inverse model by using the measured field data.

For a perfect electric conductor (PEC) target located in the observation area with the boundary ∂Ω, the potential of each point in region Ω is φ. According to the electrostatic field theory [25], the base Poisson equation with boundary conditions is considered, which are shown in (1), (2) and (3):

(1) $$\nabla^{2}\varphi =-f,\;\mathrm{in}\;\mathrm{region}\;\Omega ,$$

(2) $$\varphi =\varphi_{s},\;\mathrm{on}\;\mathrm{boundary}\;\partial \Omega ,$$

(3) ∇·ρ=0, on boundary ∂Ω,

where *f* is the source distribution in region Ω, $\rho =\frac{\partial \varphi }{\partial n}$, and n is the norm vector on boundary surface ∂Ω pointing to the region Ω. According to Green’s function, the test function in three-dimensional space is given in (4):

(4) $$\left\{\begin{array}{c} W=\frac{1}{4\pi r}\\ \frac{\partial W}{\partial n}=-\frac{1}{4\pi r^{2}}\frac{\partial r}{\partial n}\\ r=\left|r-r^{\prime }\right| \end{array},\right.$$

where r is the position of the observation point and $r^{\prime }$ is the position of the source point. Thus, the potential φ on boundary surface ∂Ω can be written as:

(5) $${}_{p.v.}\int_{\partial \Omega }W\rho ds-\varphi_{s}\left(\frac{1}{2}+{}_{p.v.}\int_{\partial \Omega }\frac{\partial W}{\partial n}ds\right)=\int_{\Omega }-fWdv,$$

where p.v. denotes Cauchy’s principal value integration. According to (3), we have:

(6) $$\int_{\partial \Omega }\rho ds=0.$$

In order to solve the equation, we discretize the boundary surface into *N* triangular patches. Thus, we have:

(7) $$\sum_{i=1,i\neq j}^{N}W_{ji}\rho_{i}\Delta s_{i}+\varphi_{s}\left(-\frac{1}{2}-\sum_{i=1,i\neq j}^{N}\frac{\partial W_{ji}}{\partial n_{i}}\Delta s_{i}\right)=\int_{\Omega }-fW_{jp}dv,$$

(8) $$\sum_{i=1}^{N}\rho_{i}\Delta s_{i}=0,$$

(9) $$\left\{\begin{array}{c} W_{ji}=\frac{1}{4\pi r_{ji}}\\ \frac{\partial W_{ji}}{\partial n_{i}}=-\frac{1}{4\pi r_{ji}^{2}}\frac{\partial r_{ji}}{\partial n_{i}}\\ r_{ji}=\left|r_{j}-r_{i}\right| \end{array},\right.$$

where i,j=1,2,3,…,N, $\Delta s_{i}$ denotes the *i*-th triangular patch of ∂Ω with the norm vector $n_{i}$ and σ represents the conductivity of the material in the localization region. In this paper, the dipole source is used as the emitter. Thus, we define $r_{p}$ as the position of the dipole source, and p is the dipole moment of the dipole source. Thus, we have:

(10) $$Z_{s}^{s}=\left[\begin{array}{ccccc} 0 & W_{12}\Delta s_{2} & \cdots & W_{1N}\Delta s_{N} & -\frac{1}{2}-\sum_{i=2}^{N}\frac{\partial W_{1i}}{\partial n_{i}}\Delta s_{i}\\ W_{21}\Delta s_{1} & 0 & \cdots & W_{2N}\Delta s_{N} & -\frac{1}{2}-\sum_{i=1,i\neq 2}^{N}\frac{\partial W_{2i}}{\partial n_{i}}\Delta s_{i}\\ & & \cdots & & \\ W_{N1}\Delta s_{1} & W_{N2}\Delta s_{2} & \cdots & 0 & -\frac{1}{2}-\sum_{i=1,i\neq j}^{N}\frac{\partial W_{Ni}}{\partial n_{i}}\Delta s_{i}\\ \Delta s_{1} & \Delta s_{2} & \cdots & \Delta s_{N} & 0 \end{array}\right],$$

(11) $$Y={\left[\rho_{1}\;\rho_{2}\;\cdots \;\rho_{N}\;\varphi_{s}\right]}^{T},$$

(12) $$G_{p}^{s}=\frac{1}{4\pi \sigma }{\left[\begin{array}{c} \begin{array}{ccccc} \frac{e_{x}\cdot \left(r_{1}-r_{p}\right)}{{\left|r_{1}-r_{p}\right|}^{3}} & \frac{e_{x}\cdot \left(r_{2}-r_{p}\right)}{{\left|r_{2}-r_{p}\right|}^{3}} & \cdots & \frac{e_{x}\cdot \left(r_{N}-r_{p}\right)}{{\left|r_{N}-r_{p}\right|}^{3}} & 0 \end{array}\\ \begin{array}{ccccc} \frac{e_{y}\cdot \left(r_{1}-r_{p}\right)}{{\left|r_{1}-r_{p}\right|}^{3}} & \frac{e_{y}\cdot \left(r_{2}-r_{p}\right)}{{\left|r_{2}-r_{p}\right|}^{3}} & \cdots & \frac{e_{y}\cdot \left(r_{N}-r_{p}\right)}{{\left|r_{N}-r_{p}\right|}^{3}} & 0 \end{array}\\ \begin{array}{ccccc} \frac{e_{z}\cdot \left(r_{1}-r_{p}\right)}{{\left|r_{1}-r_{p}\right|}^{3}} & \frac{e_{z}\cdot \left(r_{2}-r_{p}\right)}{{\left|r_{2}-r_{p}\right|}^{3}} & \cdots & \frac{e_{z}\cdot \left(r_{N}-r_{p}\right)}{{\left|r_{N}-r_{p}\right|}^{3}} & 0 \end{array} \end{array}\right]}^{T},$$

(13) $$p={\left[\begin{array}{ccc} p_{x} & p_{y} & p_{z} \end{array}\right]}^{T}.$$

Then, the matrix equation resulting from (7) can be explicitly written as:

(14) $$Z_{s}^{s}{Y=G}_{p}^{s}p,$$

where Y denotes the unknowns, $G_{p}^{s}$ represents the excitation of the electric dipole source on the boundary surface, ${\left(\cdot \right)}^{T}$ denotes the transpose operation and $e_{x}$, $e_{y}$ and $e_{z}$ are the unit vectors in the *x*, *y* and *z* directions, respectively. By solving the matrix equation, the unknowns Y are obtained:

(15) $$Y={\left(Z_{s}^{s}\right)}^{-1}G_{p}^{s}p.$$

In order to measure the potential in the locating area Ω, we assume that (K+1) electrodes are set at points $r_{k}^{\mathrm{rec}}$ in the water, which act as the receiving antenna where *k* = 1, 2, 3, *…*, *K* + 1. Additionally, we refer to the (K+1)-th electrode as reference point. The potential of each receptor can be denoted as follows:

(16) $$\varphi_{k}^{\mathrm{rec}}=\int_{\Omega }fW^{\mathrm{rec}}dv+\int_{\partial \Omega }W^{\mathrm{rec}}\rho ds-\varphi_{s}\int_{\partial \Omega }\frac{\partial W^{\mathrm{rec}}}{\partial n}ds.$$

We then discretize (16), yielding:

(17) $$\varphi_{k}^{\mathrm{rec}}=\int_{\Omega }fW_{kp}^{\mathrm{rec}}dv+\sum_{i=1}^{N}W_{ki}^{\mathrm{rec}}\rho_{i}\Delta s_{i}-\varphi_{s}\sum_{i=1}^{N}\frac{\partial W_{ki}^{\mathrm{rec}}}{\partial n_{i}}\Delta s_{i}.$$

The matrix expression is obtained:

(18) $$\Psi =G_{p}p+Z_{s}{\left(Z_{s}^{s}\right)}^{-1}G_{p}^{s}p,$$

(19) $$\Psi_{\mathrm{ref}}=G_{p}^{\mathrm{ref}}p+Z_{s}^{\mathrm{ref}}{\left(Z_{s}^{s}\right)}^{-1}G_{p}^{s}p,$$

where:

(20) $$\Psi ={\left[\varphi_{1}^{\mathrm{rec}}\;\varphi_{2}^{\mathrm{rec}}\;\cdots \;\varphi_{K}^{\mathrm{rec}}\right]}^{T},$$

(21) $$G_{p}=-\frac{1}{4\pi \sigma }{\left[\begin{array}{c} \begin{array}{cccc} \frac{e_{x}\cdot \left(r_{1}^{\mathrm{rec}}-r_{p}\right)}{{\left|r_{1}^{\mathrm{rec}}-r_{p}\right|}^{3}} & \frac{e_{x}\cdot \left(r_{2}^{\mathrm{rec}}-r_{p}\right)}{{\left|r_{2}^{\mathrm{rec}}-r_{p}\right|}^{3}} & \cdots & \frac{e_{x}\cdot \left(r_{K}^{\mathrm{rec}}-r_{p}\right)}{{\left|r_{K}^{\mathrm{rec}}-r_{p}\right|}^{3}} \end{array}\\ \begin{array}{cccc} \frac{e_{y}\cdot \left(r_{1}^{\mathrm{rec}}-r_{p}\right)}{{\left|r_{1}^{\mathrm{rec}}-r_{p}\right|}^{3}} & \frac{e_{y}\cdot \left(r_{2}^{\mathrm{rec}}-r_{p}\right)}{{\left|r_{2}^{\mathrm{rec}}-r_{p}\right|}^{3}} & \cdots & \frac{e_{y}\cdot \left(r_{K}^{\mathrm{rec}}-r_{p}\right)}{{\left|r_{K}^{\mathrm{rec}}-r_{p}\right|}^{3}} \end{array}\\ \begin{array}{cccc} \frac{e_{z}\cdot \left(r_{1}^{\mathrm{rec}}-r_{p}\right)}{{\left|r_{1}^{\mathrm{rec}}-r_{p}\right|}^{3}} & \frac{e_{z}\cdot \left(r_{2}^{\mathrm{rec}}-r_{p}\right)}{{\left|r_{2}^{\mathrm{rec}}-r_{p}\right|}^{3}} & \cdots & \frac{e_{z}\cdot \left(r_{K}^{\mathrm{rec}}-r_{p}\right)}{{\left|r_{K}^{\mathrm{rec}}-r_{p}\right|}^{3}} \end{array} \end{array}\right]}^{T},$$

(22) $$G_{p}^{\mathrm{ref}}=-\frac{1}{4\pi \sigma }{\left[\begin{array}{c} 1\\ 1\\ \vdots \\ 1 \end{array}\right]}_{K\times 1}\left[\begin{array}{ccc} \frac{e_{x}\cdot \left(r_{K+1}^{\mathrm{rec}}-r_{p}\right)}{{\left|r_{K+1}^{\mathrm{rec}}-r_{p}\right|}^{3}} & \frac{e_{y}\cdot \left(r_{K+1}^{\mathrm{rec}}-r_{p}\right)}{{\left|r_{K+1}^{\mathrm{rec}}-r_{p}\right|}^{3}} & \frac{e_{z}\cdot \left(r_{K+1}^{\mathrm{rec}}-r_{p}\right)}{{\left|r_{K+1}^{\mathrm{rec}}-r_{p}\right|}^{3}} \end{array}\right],$$

(23) $$Z_{s}=\left[\begin{array}{ccccc} W_{11}^{\mathrm{rec}}\Delta s_{1} & W_{12}^{\mathrm{rec}}\Delta s_{2} & \cdots & W_{1N}^{\mathrm{rec}}\Delta s_{N} & -\sum_{i=1}^{N}\frac{\partial W_{1i}^{\mathrm{rec}}}{\partial n_{i}}\Delta s_{i}\\ W_{21}^{\mathrm{rec}}\Delta s_{1} & W_{22}^{\mathrm{rec}}\Delta s_{2} & \cdots & W_{2N}^{\mathrm{rec}}\Delta s_{N} & -\sum_{i=1}^{N}\frac{\partial W_{2i}^{\mathrm{rec}}}{\partial n_{i}}\Delta s_{i}\\ & & \cdots & & \\ W_{K1}^{\mathrm{rec}}\Delta s_{1} & W_{K2}^{\mathrm{rec}}\Delta s_{2} & \cdots & W_{KN}^{\mathrm{rec}}\Delta s_{N} & -\sum_{i=1}^{N}\frac{\partial W_{Ki}^{\mathrm{rec}}}{\partial n_{i}}\Delta s_{i} \end{array}\right],$$

(24) $$Z_{s}^{\mathrm{ref}}={\left[\begin{array}{c} 1\\ 1\\ \vdots \\ 1 \end{array}\right]}_{K\times 1}\left[\begin{array}{ccccc} W_{(K+1)1}^{\mathrm{rec}}\Delta s_{1} & W_{(K+1)2}^{\mathrm{rec}}\Delta s_{2} & \cdots & W_{(K+1)N}^{\mathrm{rec}}\Delta s_{N} & -\sum_{i=1}^{N}\frac{\partial W_{(K+1)i}^{\mathrm{rec}}}{\partial n_{i}}\Delta s_{i} \end{array}\right],$$

(25) $$\left\{\begin{array}{c} W_{ki}^{\mathrm{rec}}=\frac{1}{4\pi r_{ki}}\\ \frac{\partial W_{ki}^{\mathrm{rec}}}{\partial n_{i}}=-\frac{1}{4\pi r_{ki}^{2}}\frac{\partial r_{ki}}{\partial n_{i}}\\ r_{ki}=\left|r_{k}^{\mathrm{rec}}-r_{i}\right| \end{array}.\right.$$

For the dielectric target, the matrices $Z_{s}^{s}$, Y, $G_{p}^{s}$, $G_{p}$, $G_{p}^{\mathrm{ref}}$, $Z_{s}$ and $Z_{s}^{\mathrm{ref}}$ have different expressions because of the boundary condition, ρ=0. Thus, the expression of matrices is directly given by:

(26) $$Z_{s}^{s}=\left[\begin{array}{ccccc} \frac{1}{2} & \frac{\partial W_{12}}{\partial n_{2}}\Delta s_{2} & \frac{\partial W_{13}}{\partial n_{3}}\Delta s_{3} & \cdots & \frac{\partial W_{1N}}{\partial n_{N}}\Delta s_{N}\\ \frac{\partial W_{21}}{\partial n_{1}}\Delta s_{1} & \frac{1}{2} & \frac{\partial W_{23}}{\partial n_{3}}\Delta s_{3} & \cdots & \frac{\partial W_{2N}}{\partial n_{N}}\Delta s_{N}\\ \frac{\partial W_{31}}{\partial n_{1}}\Delta s_{1} & \frac{\partial W_{32}}{\partial n_{2}}\Delta s_{2} & \frac{1}{2} & \cdots & \frac{\partial W_{3N}}{\partial n_{N}}\Delta s_{N}\\ & & & \cdots & \\ \frac{\partial W_{N1}}{\partial n_{1}}\Delta s_{1} & \frac{\partial W_{N2}}{\partial n_{2}}\Delta s_{2} & \frac{\partial W_{N3}}{\partial n_{3}}\Delta s_{3} & \cdots & \frac{1}{2} \end{array}\right],$$

(27) $$Y={\left[\varphi_{1}\;\varphi_{2}\;\cdots \;\varphi_{N}\right]}^{T},$$

(28) $$G_{p}^{s}=\frac{1}{4\pi \sigma }{\left[\begin{array}{c} \begin{array}{cccc} \frac{e_{x}\cdot \left(r_{1}-r_{p}\right)}{{\left|r_{1}-r_{p}\right|}^{3}} & \frac{e_{x}\cdot \left(r_{2}-r_{p}\right)}{{\left|r_{2}-r_{p}\right|}^{3}} & \cdots & \frac{e_{x}\cdot \left(r_{N}-r_{p}\right)}{{\left|r_{N}-r_{p}\right|}^{3}} \end{array}\\ \begin{array}{cccc} \frac{e_{y}\cdot \left(r_{1}-r_{p}\right)}{{\left|r_{1}-r_{p}\right|}^{3}} & \frac{e_{y}\cdot \left(r_{2}-r_{p}\right)}{{\left|r_{2}-r_{p}\right|}^{3}} & \cdots & \frac{e_{y}\cdot \left(r_{N}-r_{p}\right)}{{\left|r_{N}-r_{p}\right|}^{3}} \end{array}\\ \begin{array}{cccc} \frac{e_{z}\cdot \left(r_{1}-r_{p}\right)}{{\left|r_{1}-r_{p}\right|}^{3}} & \frac{e_{z}\cdot \left(r_{2}-r_{p}\right)}{{\left|r_{2}-r_{p}\right|}^{3}} & \cdots & \frac{e_{z}\cdot \left(r_{N}-r_{p}\right)}{{\left|r_{N}-r_{p}\right|}^{3}} \end{array} \end{array}\right]}^{T},$$

(29) $$G_{p}=\frac{1}{4\pi \sigma }{\left[\begin{array}{c} \begin{array}{cccc} \frac{e_{x}\cdot \left(r_{1}^{\mathrm{rec}}-r_{p}\right)}{{\left|r_{1}^{\mathrm{rec}}-r_{p}\right|}^{3}} & \frac{e_{x}\cdot \left(r_{2}^{\mathrm{rec}}-r_{p}\right)}{{\left|r_{2}^{\mathrm{rec}}-r_{p}\right|}^{3}} & \cdots & \frac{e_{x}\cdot \left(r_{K}^{\mathrm{rec}}-r_{p}\right)}{{\left|r_{K}^{\mathrm{rec}}-r_{p}\right|}^{3}} \end{array}\\ \begin{array}{cccc} \frac{e_{y}\cdot \left(r_{1}^{\mathrm{rec}}-r_{p}\right)}{{\left|r_{1}^{\mathrm{rec}}-r_{p}\right|}^{3}} & \frac{e_{y}\cdot \left(r_{2}^{\mathrm{rec}}-r_{p}\right)}{{\left|r_{2}^{\mathrm{rec}}-r_{p}\right|}^{3}} & \cdots & \frac{e_{y}\cdot \left(r_{K}^{\mathrm{rec}}-r_{p}\right)}{{\left|r_{K}^{\mathrm{rec}}-r_{p}\right|}^{3}} \end{array}\\ \begin{array}{cccc} \frac{e_{z}\cdot \left(r_{1}^{\mathrm{rec}}-r_{p}\right)}{{\left|r_{1}^{\mathrm{rec}}-r_{p}\right|}^{3}} & \frac{e_{z}\cdot \left(r_{2}^{\mathrm{rec}}-r_{p}\right)}{{\left|r_{2}^{\mathrm{rec}}-r_{p}\right|}^{3}} & \cdots & \frac{e_{z}\cdot \left(r_{K}^{\mathrm{rec}}-r_{p}\right)}{{\left|r_{K}^{\mathrm{rec}}-r_{p}\right|}^{3}} \end{array} \end{array}\right]}^{T},$$

(30) $$G_{p}^{\mathrm{ref}}=\frac{1}{4\pi \sigma }{\left[\begin{array}{c} 1\\ 1\\ \vdots \\ 1 \end{array}\right]}_{K\times 1}\left[\begin{array}{ccc} \frac{e_{x}\cdot \left(r_{K+1}^{\mathrm{rec}}-r_{p}\right)}{{\left|r_{K+1}^{\mathrm{rec}}-r_{p}\right|}^{3}} & \frac{e_{y}\cdot \left(r_{K+1}^{\mathrm{rec}}-r_{p}\right)}{{\left|r_{K+1}^{\mathrm{rec}}-r_{p}\right|}^{3}} & \frac{e_{z}\cdot \left(r_{K+1}^{\mathrm{rec}}-r_{p}\right)}{{\left|r_{K+1}^{\mathrm{rec}}-r_{p}\right|}^{3}} \end{array}\right],$$

(31) $$Z_{s}=-\left[\begin{array}{ccccc} \frac{\partial W_{11}^{\mathrm{rec}}}{\partial n_{1}}\Delta s_{1} & \frac{\partial W_{12}^{\mathrm{rec}}}{\partial n_{2}}\Delta s_{2} & \cdots & \frac{\partial W_{1N}^{\mathrm{rec}}}{\partial n_{N}}\Delta s_{N}\\ \frac{\partial W_{21}^{\mathrm{rec}}}{\partial n_{1}}\Delta s_{1} & \frac{\partial W_{22}^{\mathrm{rec}}}{\partial n_{2}}\Delta s_{2} & \cdots & \frac{\partial W_{2N}^{\mathrm{rec}}}{\partial n_{N}}\Delta s_{N}\\ & & \cdots & & \\ \frac{\partial W_{K1}^{\mathrm{rec}}}{\partial n_{1}}\Delta s_{1} & \frac{\partial W_{K2}^{\mathrm{rec}}}{\partial n_{2}}\Delta s_{2} & \cdots & \frac{\partial W_{KN}^{\mathrm{rec}}}{\partial n_{N}}\Delta s_{N} \end{array}\right],$$

(32) $$Z_{s}^{\mathrm{ref}}=-{\left[\begin{array}{c} 1\\ 1\\ \vdots \\ 1 \end{array}\right]}_{K\times 1}\left[\begin{array}{cccc} \frac{\partial W_{(K+1)1}^{\mathrm{rec}}}{\partial n_{1}}\Delta s_{1} & \frac{\partial W_{(K+1)2}^{\mathrm{rec}}}{\partial n_{2}}\Delta s_{2} & \cdots & \frac{\partial W_{(K+1)N}^{\mathrm{rec}}}{\partial n_{N}}\Delta s_{N} \end{array}\right],$$

Now, we obtain the potential matrix expression for a PEC target or dielectric target in the underwater environment with a dipole source. The voltage between the receiving electrodes and the reference point can be represented as:

(33) $$\Phi =\Psi -\Psi_{\mathrm{ref}}=G_{1}p+G_{2}p,$$

where:

(34) $$G_{1}=G_{p}-G_{p}^{\mathrm{ref}},$$

(35) $$G_{2}=\left(Z_{s}-Z_{s}^{\mathrm{ref}}\right){\left(Z_{s}^{s}\right)}^{-1}G_{p}^{s}.$$

It can be seen from (33) that $G_{1}$ and $G_{2}$ are the gain matrices [26], which correspond to the dipole source of the receiving antenna and the induced field of the target, respectively. From (34) and (35), it can be seen that only $G_{2}$ contains the position information of the target. The manifold $G_{1}$ has no effects on the locating results, which can be regarded as the redundant item. In order to reduce the influence of $G_{1}$, an improved UCA system is designed, in which the electrodes are set at the equipotential points for a given electric dipole source. As a result, the energy that the UCA system received from the electric dipole source ${∥G_{1}p∥}_{F}^{2}$ would be zero in the ideal situation. Thus, Equation (33) is simplified as:

(36) $$\Phi =G_{2}p.$$

We refer to (36) as the BEM model. It should be noted that the prior information, the radius of the spherical target, should be known before locating by using the BEM model. As the UCA can only sense the field induced by the target in the observation area, the target in the BEM model can be simplified as an equivalent dipole source. Hence, we also have the simplified model:

(37) $$\Phi =G_{3}p^{\prime }=\left(G_{t}-G_{t}^{\mathrm{ref}}\right)p^{\prime },$$

(38) $$G_{t}=\frac{1}{4\pi \sigma }{\left[\begin{array}{c} \begin{array}{cccc} \frac{e_{x}\cdot \left(r_{1}^{\mathrm{rec}}-r_{t}\right)}{{\left|r_{1}^{\mathrm{rec}}-r_{t}\right|}^{3}} & \frac{e_{x}\cdot \left(r_{2}^{\mathrm{rec}}-r_{t}\right)}{{\left|r_{2}^{\mathrm{rec}}-r_{t}\right|}^{3}} & \cdots & \frac{e_{x}\cdot \left(r_{K}^{\mathrm{rec}}-r_{t}\right)}{{\left|r_{K}^{\mathrm{rec}}-r_{t}\right|}^{3}} \end{array}\\ \begin{array}{cccc} \frac{e_{y}\cdot \left(r_{1}^{\mathrm{rec}}-r_{t}\right)}{{\left|r_{1}^{\mathrm{rec}}-r_{t}\right|}^{3}} & \frac{e_{y}\cdot \left(r_{2}^{\mathrm{rec}}-r_{t}\right)}{{\left|r_{2}^{\mathrm{rec}}-r_{t}\right|}^{3}} & \cdots & \frac{e_{y}\cdot \left(r_{K}^{\mathrm{rec}}-r_{t}\right)}{{\left|r_{K}^{\mathrm{rec}}-r_{t}\right|}^{3}} \end{array}\\ \begin{array}{cccc} \frac{e_{z}\cdot \left(r_{1}^{\mathrm{rec}}-r_{t}\right)}{{\left|r_{1}^{\mathrm{rec}}-r_{t}\right|}^{3}} & \frac{e_{z}\cdot \left(r_{2}^{\mathrm{rec}}-r_{t}\right)}{{\left|r_{2}^{\mathrm{rec}}-r_{t}\right|}^{3}} & \cdots & \frac{e_{z}\cdot \left(r_{K}^{\mathrm{rec}}-r_{t}\right)}{{\left|r_{K}^{\mathrm{rec}}-r_{t}\right|}^{3}} \end{array} \end{array}\right]}^{T},$$

(39) $$G_{t}^{\mathrm{ref}}=\frac{1}{4\pi \sigma }{\left[\begin{array}{c} 1\\ 1\\ \vdots \\ 1 \end{array}\right]}_{K\times 1}\left[\begin{array}{ccc} \frac{e_{x}\cdot \left(r_{K+1}^{\mathrm{rec}}-r_{t}\right)}{{\left|r_{K+1}^{\mathrm{rec}}-r_{t}\right|}^{3}} & \frac{e_{y}\cdot \left(r_{K+1}^{\mathrm{rec}}-r_{t}\right)}{{\left|r_{K+1}^{\mathrm{rec}}-r_{t}\right|}^{3}} & \frac{e_{z}\cdot \left(r_{K+1}^{\mathrm{rec}}-r_{t}\right)}{{\left|r_{K+1}^{\mathrm{rec}}-r_{t}\right|}^{3}} \end{array}\right],$$

where $r_{t}$ is the position of the equivalent dipole source. $p^{\prime }$ denotes the dipole moment of the equivalent dipole source. It can be seen that the simplified model does not need the prior information, the radius of the spherical target, which is more flexible for estimating the position of the target. However, the simplified model would result in higher locating error because it is an equivalent model.

### 2.2. Localization Based on the MP-MUSIC Algorithm

In order to estimate the position of the target, the MP-MUSIC algorithm is introduced, which is a kind of subspace-based, high-resolution locating algorithm [23,27]. The polarization of the incident signals does not need to be known, which is suitable for underwater target locating. For the canonical MUSIC algorithm in direction-of-arrival (DOA) estimation, it is mainly used to estimate the one-dimensional direction of the arriving wave, in which the phase of the wave is the main argument of the array manifold. Different from the canonical MUSIC algorithm, in this paper, we estimate the three-dimensional position of the target based on the voltage amplitude in each channel in the UCA antenna, which means that the gain matrix or the array manifold is associated with the voltage amplitude. The electric dipole source in the UCA system is associated with direct current (DC) excitation, since the electric dipole source is a controllable source. We assume that the orientation of the UCA system is quasi-static to the target during the measurements. During the locating process, the localization system should measure the voltage between the receiving electrodes and the reference point with *M* snapshots. Thus, the data in (36) and (37) are acquired by:

(40) Φ(t)=Gp(t)+e(t)

where Φ(t) and e(t) are K×M dimensional signal and noise matrices. The noise matrix e(t) is assumed to be zero mean with covariance of $E\left\{e_{t}e_{t}^{H}\right\}={\sigma_{e}}^{2}I$, where $E\left\{\cdot \right\}$ denotes the expected value of the argument, ${\left(\cdot \right)}^{H}$ is the Hermitian transpose operator and I denotes the identity matrix. p(t) is a 3×M matrix. First, MP-MUSIC estimates the array covariance matrix Φ(t) under the zero-mean white noise assumption:

(41) $$R_{\Phi }=E\left\{\Phi (t)\Phi {\left(t\right)}^{H}\right\}=GE\left\{p(t)p{\left(t\right)}^{H}\right\}G^{H}+{\sigma_{e}}^{2}I.$$

$R_{\Phi }$ is a Hermitian matrix of full rank *K*. By using eigen decomposition, we have:

(42) $$R_{\Phi }=U\Sigma U^{H}.$$

where Σ is the diagonal matrix with the eigenvalues $\lambda_{1}\geq \lambda_{2}\geq \cdots \geq \lambda_{K}\geq 0$. U are the corresponding eigenvectors. According to the MP-MUSIC principle and the number of targets in this paper, the vector space spanned by the first eigenvector is the signal subspace $U_{S}$. The vector space spanned by the last (K−1) eigenvectors is defined as the noise subspace $U_{N}$. Thus, we have $U=\left[\begin{array}{cc} U_{S} & U_{N} \end{array}\right]$. The projection matrix of the noise subspace can be written as $P^{⊥}=I-U_{S}U_{S}^{H}$. Then, the MP-MUSIC spectrum can be represented as:

(43) $$P=\frac{1}{\lambda_{\min}\left(G^{H}P^{⊥}G,G^{H}G\right)}$$

where $\lambda_{\min}\left(\cdot \right)$ indicates the minimum eigenvalue. According to the MUSIC theory, the target can be located by finding out the peak of the spectrum *P*.

According to [23,26,28], we give the algorithmic steps for locating the target from the original measured voltage data based on the BEM model:

- Step 1: Discretize the boundary of the target and calculate the boundary matrix $Z_{s}^{s}$ according to the prior information about the target. By inversing the boundary matrix, we have ${\left(Z_{s}^{s}\right)}^{-1}$.
- Step 2: Measure the voltage by using the UCA; the matrix Φ(t) is formed with the size of K×M.
- Step 3: According to (41), the covariance matrix $R_{\Phi }$ can be constructed.
- Step 4: Obtain the eigenvalues $\lambda_{i}$ of the matrix $R_{\Phi }$ via eigen decomposition, which are arranged in decreasing order, $\lambda_{1}\geq \lambda_{2}\geq \cdots \geq \lambda_{K}\geq 0$.
- Step 5: Obtain the required signal subspace $U_{S}$, which is the eigenvector corresponding to the maximum eigenvalue $\lambda_{1}$.
- Step 6: Calculate the orthogonal projector for $U_{S}$ as $P^{⊥}=I-U_{S}U_{S}^{H}$.
- Step 7: Scan the observation area where the target exists with a series locating hypothesis $r_{t1},r_{t2},\cdots ,r_{\mathrm{tL}}$.
- Step 8: According to (35), calculate the matrix $G_{2(i)}$ for each spatial points $r_{ti}$, i=1,2,⋯,L.
- Step 9: Obtain the spectrum $P_{\left(i\right)}=\frac{1}{\lambda_{\min}(G_{2(i)}^{H}P^{⊥}G_{2(i)},G_{2(i)}^{H}G_{2(i)})}$ via the generalized eigen decomposition.
- Step 10: Find out the global maxima of $P_{\max}=P_{\left(j\right)}$. Then, the target position is estimated by $r_{tj}$.

In order to express the locating processes based on the MP-MUSIC algorithm more clearly, we also give the pseudocode in Algorithm 1.

**Algorithm 1** Locating the target based on the MP-MUSIC algorithm.
**Input:** $Z_{s}^{s}$, Φ(t), $r_{t1},r_{t2},\cdots ,r_{\mathrm{tL}}$;    % *input the raw data and information of the environment***Output:** $r_{\mathrm{estimate}}$;                       % *output the target estimation position*
1:$R_{\Phi }=E\left\{\Phi (t)\Phi {\left(t\right)}^{H}\right\}$;2:$\left[U,\Sigma \right]=\mathrm{eig}\left(R_{\Phi }\right)$;3:$\left[\lambda ,\mathrm{index}\_\lambda \right]=\max\left(\mathrm{diag}\left(\Sigma \right)\right)$;4:$U_{S}=U\left(:,\mathrm{index}\_\lambda \right)$;5:$P^{⊥}=I-U_{S}U_{S}^{H}$;6:**for** i=1 to L **do**7:   $G_{2(i)}=G_{2}\left(r_{ti}\right)$;8:   $\left[V,D\right]=\mathrm{eig}\left(G_{2(i)}^{H}P^{⊥}G_{2(i)},G_{2(i)}^{H}G_{2(i)}\right)$;9:   $P_{\left(i\right)}=\frac{1}{\min\left(\mathrm{diag}(D)\right)}$;10:**end for**11:$\left[P_{\max},j\right]=\max\left(P\right)$;12:$r_{\mathrm{estimate}}=r_{tj}$;


The locating steps based on simplified model are similar to the BEM model. As the locating method based on simplified model does not need the prior information of the target, Step 1 in BEM model can be neglected, and the gain matrix $G_{2(i)}$ in Step 8 and Step 9 is replaced by $G_{3(i)}$ according to (37).

In this section, the locating methods based on the BEM model and simplified model are given in detail by using the MUSIC algorithm. From previous analysis, the locating method based on the BEM model needs prior information, which can accurately describe the induction field of the target. The locating method based on the simplified model is an equivalent model, which considers the induction field of a dipole that is close to the position of the target, making it easier to implement.

## 3. Numerical Examples

**Figure 2:**
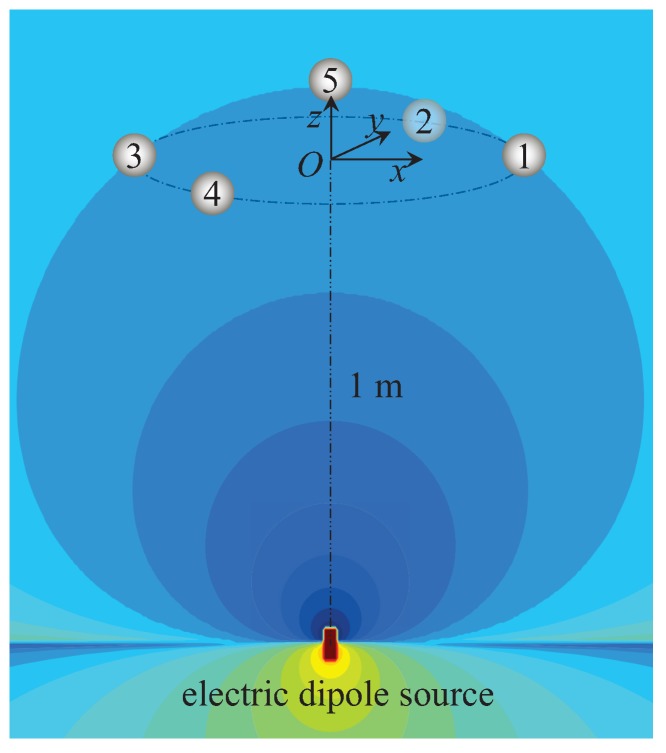
The improved UCA system with the electrodes being settled at the equipotential surface.

In this section, we present a numerical example and a simulation model to illustrate the features of our proposed localization method. The conductivity of the locating area is σ=4S/m, which is the same as the seawater. A UCA system is designed, which consists of two parts. The first part is the emitter, which is an ideal electric dipole source. The electric dipole source is situated at (0,0,−1) m with the dipole moment 1A·m along the *z*-axis. The second part is the receptor, which is the ideal points in this simulation model. The electrodes positions in the UCA system are listed in Table 1 and shown in Figure 2. The electrodes Index Numbers 1–4 are uniformly distributed on a circle of radius 0.1 m, and the fifth electrode is set as the reference electrode. We define the output data of channel *k* as the voltage between the *k*-th electrode and the reference electrode, k=1,2,3,4. Additionally, the five electrodes are set at the equipotential points of the electric dipole source, which means that the voltage from channel *k* should be zero when there is no target in the observation area. Then, a spherical target with a radius of 0.05 m is discretized into 3004 triangular patches. In this locating system, we hold the number of time samples constant at 200 for one locating operation, yielding M=200 in (40).

**Figure 3:**
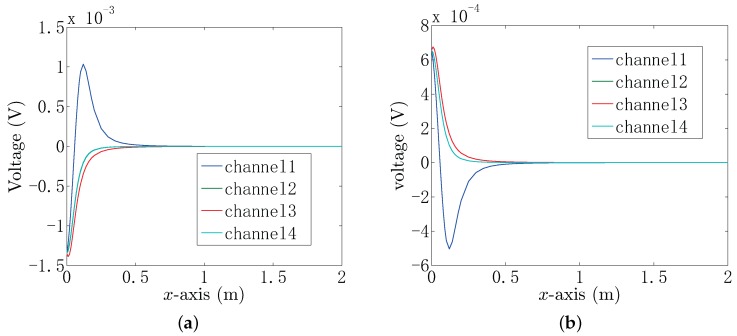
The the voltage of each channel of the UCA system when the target is set at position (x,0,0.1). (**a**) Conductor target; (**b**) insulator target.

When the target is located near the UCA system, the voltage of each channel of the UCA system can be obtained. Figure 3a shows the obtained voltage of each channel when the conductor target is set at position (x,0,0.1). Figure 3b shows the obtained voltage when the insulator target is set at position (x,0,0.1). From Figure 3, it can be seen that the voltage amplitude in Channel 1 has a different property from the other three channels. That is because the distance between the target and Electrode 1 decreases as *x* increases when 0≤x≤0.1, and it increases when x≥0.1. Electrodes 2 and 4 are symmetric about the *x*-axis, yielding that Channels 2 and 4 have the same voltage curves when the target is located on the *x*-axis. The voltage of each channel contains the position information of the target, which allows the electric-locator to estimate the target position based on the BEM and MP-MUSIC algorithm.

**Figure 4:**
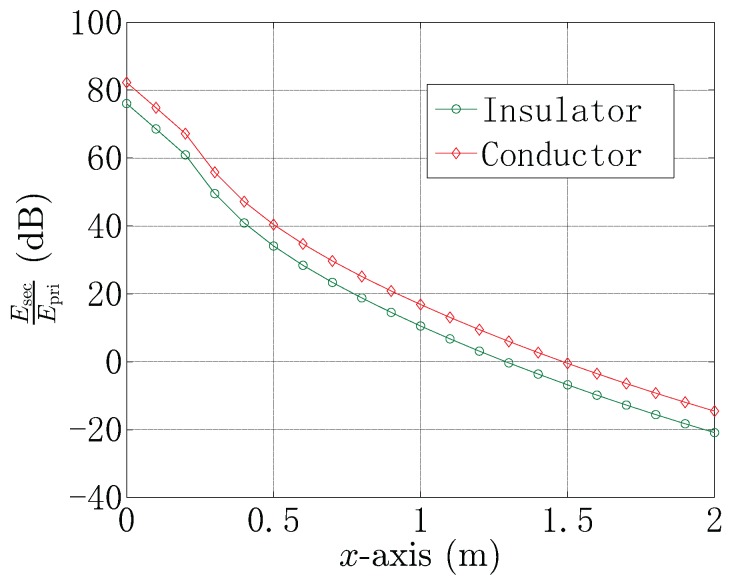
The ratio of the magnitude of the primary energy and the secondary energy $\frac{E_{\sec}}{E_{\mathrm{pri}}}$ when the target is in a different position.

The proposed UCA system can reduce the influence of the electric dipole source. Figure 4 shows the comparison between the magnitude of the primary energy generated by the emitter $E_{\mathrm{pri}}$ and the secondary energy reflected by the spherical target $E_{\sec}$. From Figure 4, it can be seen that $E_{\sec}$ plays the main role when 0≤x≤1.3 m for the conductor target and 0≤x≤1.0 m for the insulator target, where the contribution of $E_{\mathrm{pri}}$ can be neglected. However, for the conductor target, the $E_{\mathrm{pri}}$ will not be neglected, when the target lies in x>1.3 m. For the insulator target, the $E_{\mathrm{pri}}$ will not be neglected, when the target lies in x>1.0 m. In the practical scenario, the reference point is close to, but not at the equipotential point, which causes the primary energy $E_{\mathrm{pri}}$ not to be zero. Moreover, the secondary energy $E_{\sec}$ will decrease as the target gets far from the UCA, which results in the decrease of the ratio of $\frac{E_{\sec}}{E_{\mathrm{pri}}}$, where:

(44) $$E_{\mathrm{pri}}={∥G_{1}p∥}_{F}^{2},$$

(45) $$E_{\sec}={∥G_{2}p∥}_{F}^{2}.$$

**Figure 5:**
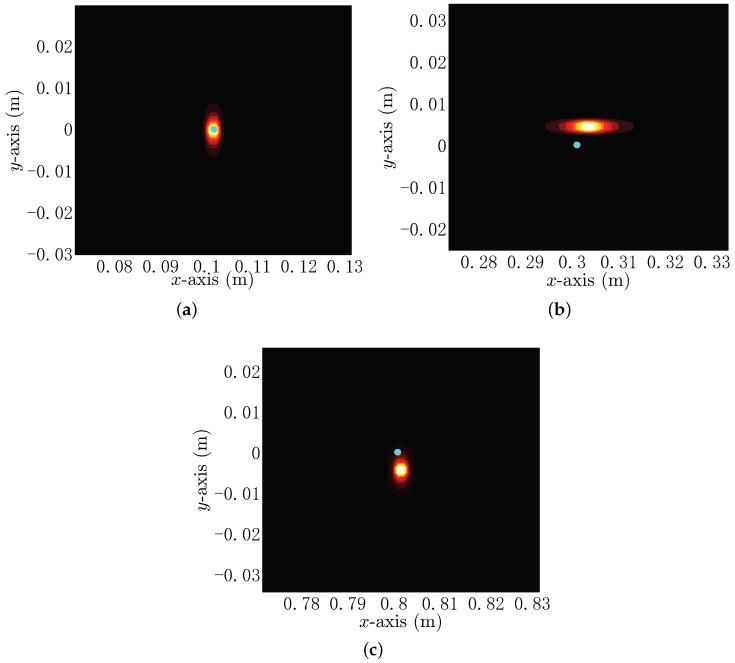
The spectrum images are given based on the MP-MUSIC for SNR=20 dB. The real position of the insulator target is marked with a blue point, and the highlighted spots in each of the spectrum images indicates the estimated position of the target. (**a**) The spectrum image of the insulator target whose true localization is (0.1,0,0.1) m; (**b**) the spectrum image of the insulator target whose true localization is (0.3,0,0.1) m; (**c**) the spectrum image of the insulator target whose true localization is (0.8,0,0.1) m.

According to the measured voltage obtained from each channel of the proposed UCA system, the spectrum can be obtained, with which the electro-locator can estimate the position of the target. The additive white Gaussian noise is added to all measured voltage data, where the squared Frobenius norm of the noiseless signal matrix ${∥Gp(t)∥}_{F}^{2}$ is one hundred times that of the squared Frobenius norm of the noise matrix ${∥e(t)∥}_{F}^{2}$. As a result, the signal-to-noise ratio (SNR) is 20 dB [29,30]. Figure 5 gives the spectrum images of an insulator with the radius of 0.05 m when it is situated at points (0.1,0,0.1) m, (0.3,0,0.1) m and (0.8,0,0.1) m, respectively. The real position of the target is marked with a blue point, and the highlighted spots in the spectrum images indicate the estimated positions of the target. The peaks of the spectrum images are (0.101,0,0.102) m, (0.303,0.005,0.096) m and (0.801,−0.004,0.089) m, which are the estimated positions by the use of the proposed electro-locator. The corresponding location errors are 0.003 m, 0.007 m and 0.012 m, which shows that the proposed locating method can be applied in underwater target locating. Next, we will study the locating performance in detail.

**Figure 6:**
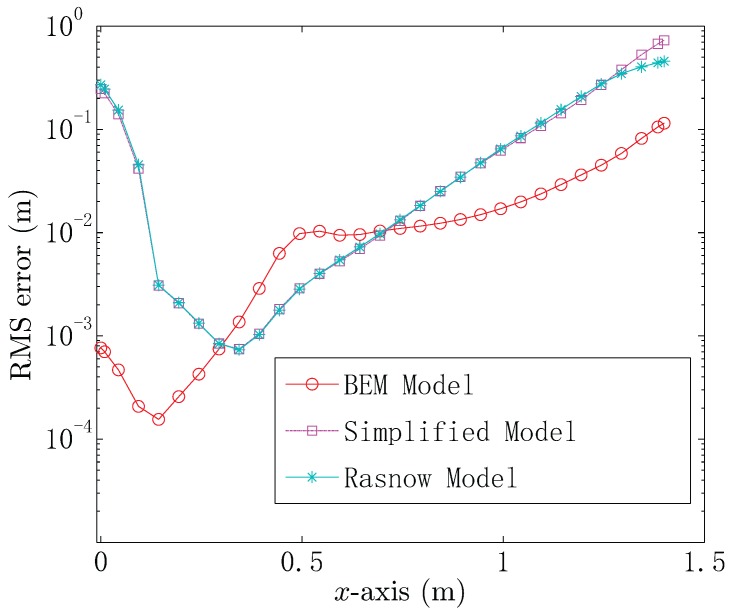
The locating RMS errors by using the boundary element method (BEM) model, the simplified model and the Rasnow model.

In the practical situation, the noise can affect the locating performance. Generally, the noise power of each channel in the UCA system is constant. Thus, we add the additive white Gaussian noise to all measured voltage data, yielding the SNR of 20 dB, when the conductor target is located at (0.8,0,0.1) m. We refer to the noise as the background noise. Figure 6 shows the root mean square (RMS) errors for locating the conductor target, which is situated at the continuous positions (x,0,0.1) m. From Figure 6, it is obvious that the simplified model has the same locating performance as the Rasnow model, when x≤1.4. The reason is that both the simplified model and the Rasnow model consider the spherical target as an equivalent electric dipole. It can also be seen that the RMS error of the three models first decreases as *x* increases. The UCA system and the conductor target are both symmetrical structures, which means that the voltage data of the four channels in the UCA system will be similar as the target gets close to the *z*-axis. As a result, the simplified model, Rasnow model and BEM model have lower locating accuracy, when the target gets close to the center of the UCA system. When the target gets far from the center of the UCA system, the localization method can give the voltage difference between each channel by using the three models, resulting in a decrease of the locating RMS error. However, the secondary energy will decrease when the target is getting far from the UCA system and the energy of the background noise remains constant. Thus, the RMS error increases when x≥0.4 for the simplified model and Rasnow model and x≥0.1 for the BEM model. It should be noted that the BEM model shows better locating performance compared with the simplified model and Rasnow model in most cases. However, the simplified model and Rasnow model provide better locating performance when 0.3≤x≤0.7.

**Figure 7:**
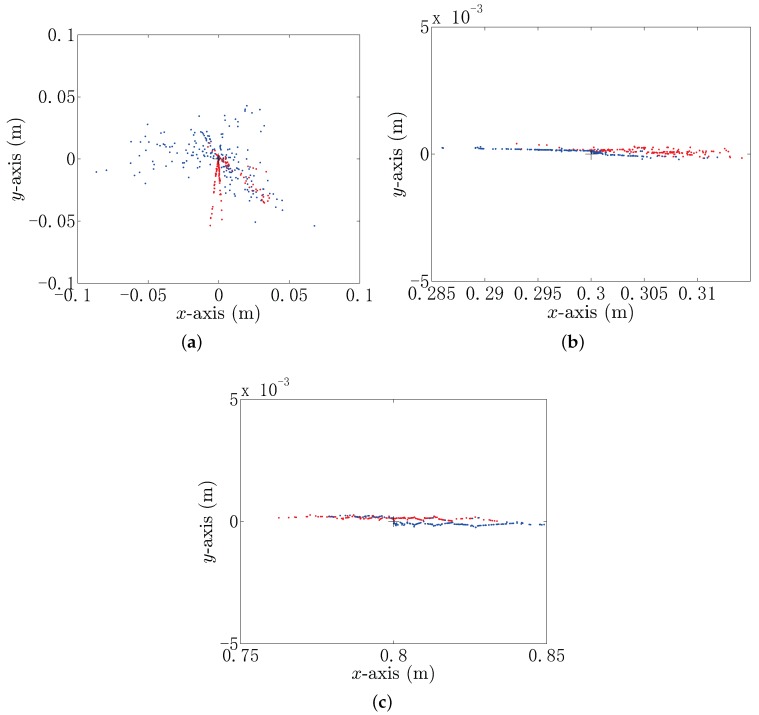
The 200-times independent locating operation by using the BEM model and the simplified model with SNR=20 dB. The black cross indicates the real position of the target. (**a**) The estimated position of the conductor target whose true localization is (0,0,0.1) m; (**b**) the estimated position of the conductor target whose true localization is (0.3,0,0.1) m; (**c**) the estimated position of the conductor target whose true localization is (0.8,0,0.1) m.

The locating results are obtained by the use of the BEM-based model and simplified model and compared under the same SNR. Figure 7 shows the 200-times independent locating operation by using the BEM model and the simplified model with SNR=20 dB when the target is situated at (0,0,0.1), (0.3,0,0.1) and (0.8,0,0.1). The blue scatters in Figure 7 are the estimated positions by using the simplified model, and the red ones are the estimated positions by using the BEM model. In Figure 7, the average estimated positions by using the simplified model are (−0.015,−0.004,0.215), (0.299,0,0.101) and (0.816,0,0.102). The average estimated positions by using the BEM model are (0.004,−0.011,0.163), (0.305,0,0.100) and (0.800,0,0.101). It can be seen from Figure 7a that the distribution of the blue scatters is sparser than that of the red scatters, but not obviously, which means that the proposed localization methods have comparable localization performance, when the target gets close to the center of the UCA system. In Figure 7b,c, we can see that the locating method based on the simplified model and BEM model provides better locating performance in the *y*-direction.

**Figure 8:**
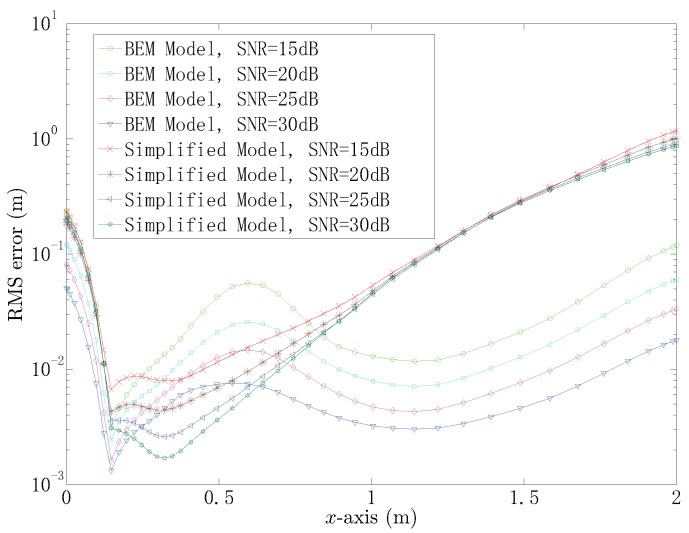
The RMS errors of the position estimated by using the simplified model and the BEM model, when the target is set at different positions.

In order to evaluate the accuracy of the proposed localization method when the target is situated at the continuous positions (x,0,0.1), the RMS errors of the estimated position by using the simplified model and BEM model at the same SNR are given and compared in Figure 8 and Table 2. For the simplified model, the RMS error curves are close to the others when x≤0.2 and x≥0.8, which means that the RMS errors would not decrease obviously as the SNR increases. The RMS errors decrease significantly as the SNR increases by using the BEM model. Furthermore, the BEM model provides more accurate estimation of the position compared with the simplified model with the same SNR when x≤0.2 and x≥0.8. It should be noted that the simplified model shows good locating performance when 0.2<x<0.8. For example, when the target is located at (0.3,0,0.1), the RMS errors are 0.008 m and 0.002 m by using the simplified model at 15 dB and 30 dB, respectively; whereas the RMS errors are 0.012 m and 0.004 m by using the BEM model.

**Figure 9:**
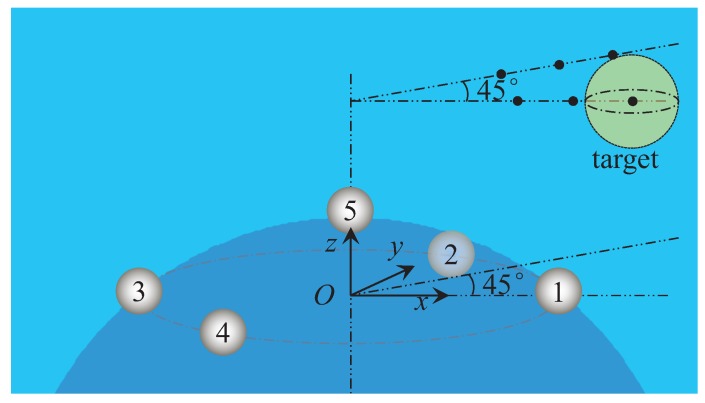
The schematic diagram of the electro-locator when the azimuths of the target are 0 deg and 45 deg.

The locating performance of the proposed UCA is investigated when the target is situated at different azimuths, which is shown in Figure 9. Two azimuths with different distances *r* from the center of the UCA are taken into consideration when the SNR is equal to 20 dB. Table 3 shows the comparison results by using the canonical MUSIC and MP-MUSIC locating algorithm, when the azimuths are zero degrees (along the $e_{x}$ direction) and 45 degrees (along the (1,1,0) direction). From Table 3, we can see that the locating method based on the BEM model and MP-MUSIC provides a similar locating performance when the azimuths are zero degrees and 45 degrees. Additionally, the locating method based on the Rasnow model and MP-MUSIC also provides a similar locating performance when the azimuths are zero degrees and 45 degrees. However the locating error significantly increases when r≥0.8 m, compared to the BEM model with the MP-MUSIC algorithm. The BEM model and canonical MUSIC algorithm-based locating method is sensitive to the azimuth, providing high locating errors when the azimuth is 45 degrees. We can also see that the Rasnow model with the canonical MUSIC algorithm could hardly give satisfactory localization when the azimuth is 45 degrees, which indicates that the Rasnow model with the canonical MUSIC algorithm is not suitable for underwater target locating. The comparison results in Table 3 indicate that the BEM model with the MP-MUSIC can satisfy the locating resolution well when the azimuth changes.

**Figure 10:**
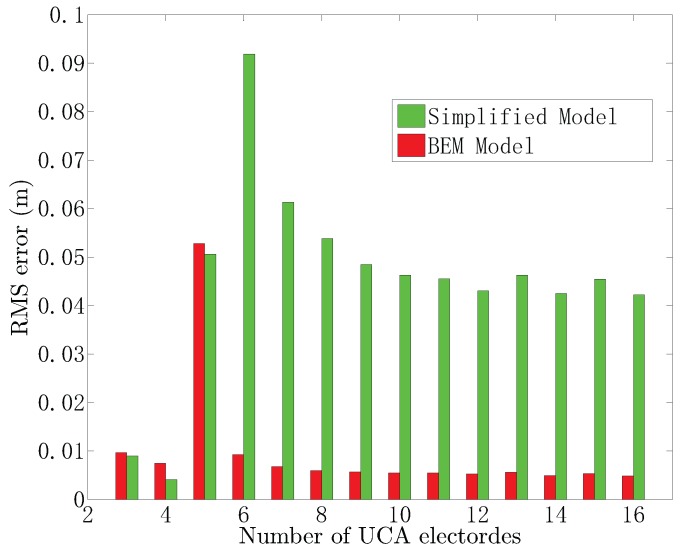
The RMS of the estimated position versus the number of electrodes.

In this paper, the receptor of the UCA system consists of five electrodes, yielding four measurement channels. Theoretically, the reference point (0,0,0.008) and points on the circle *l*: (x,y,0) are of the same potential, where $x^{2}+y^{2}=0.1^{2}$. We investigate the locating performance of the UCA system with *Q* electrodes uniformly distributed on the circle (x,y,0), Q=3,4,⋯,16. Figure 10 shows the RMS of the estimated position versus the number of electrodes by using the simplified model and BEM model, when the target is situated at (0.3,0,0.1) with SNR=20 dB. From Figure 10, we found that increasing the number of electrodes will not improve the locating accuracy significantly. It is obvious that the simplified model provides lower RMS errors compared with the BEM model when Q=3,4 and 5. However, the RMS errors are large when the number of electrodes Q≥6, and the RMS errors are about 0.5 m. The simplified model provides the best locating performance when the number of electrodes is four. For the BEM model, the RMS errors are stable as the number of electrodes increases when Q=3,4 and Q≥6. The BEM model shows the worst locating accuracy when the number of electrodes is taken as five and the RMS error is greater than 0.05 m. Thus, according to Figure 10, the optimal number of electrodes in the UCA system for the BEM model and the simplified model is four.

In this section, a locating simulation scenario is proposed to analyze the performance of the proposed localization methods and the UCA system. The simulation results show that both the BEM model and simplified model provide acceptable locating accuracy, especially when 0.1≤x≤0.8, compared with the Rasnow model. However, the proposed locating models show unsatisfactory locating results when the target gets close to the center of the UCA system. The receptor of the UCA system is specially designed, which can only sense the induction field of the target and cannot sense the field from the emitter of the UCA system itself. We also find that the MP-MUSIC provides better locating performance than the canonical MUSIC algorithm when the target is located at different azimuths. It should be noted that increasing the number of receiving electrodes would not improve the locating accuracy significantly. On the contrary, increasing the number of receiving electrodes may result in performance degradation. For this locating scenario and the UCA system, the optimal number of the electrodes on the circle *l* is four. Additionally, the gain matrix $G_{3}$ of the simplified model is much simpler than the gain matrix $G_{2}$ of the BEM model. As a result, localization based on the simplified model would reduce the computational burden. In the required real-time scenario, the simplified model would provide more advantages.

## 4. Simulation Model

**Figure 11:**
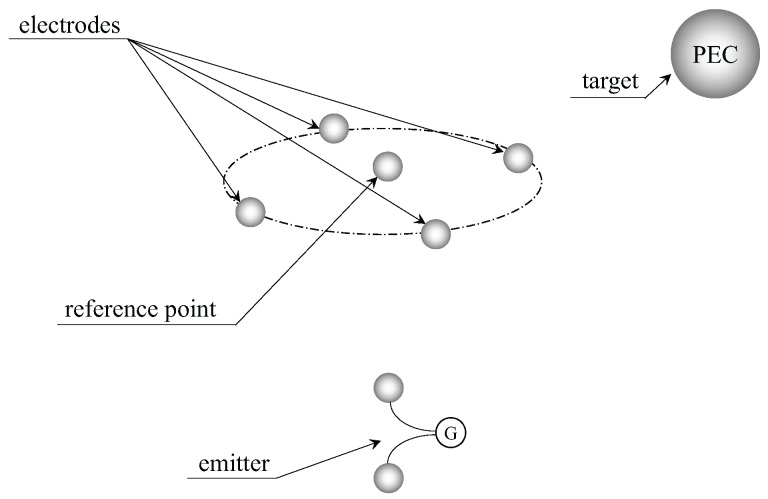
The simulation model schematic diagram implemented by using CST. PEC, perfect electric conductor.

To further verify the effectiveness of proposed localization method, a simulation model is implemented in a commercial electromagnetic simulator environment, the Computer Simulation Technology (CST) Studio Suite. By using CST, the uncertainties in a practical environment such as position error of the electrodes, the boundary effect of the water tank and position error of the target can be neglected, which would give us more objective and credible results. In this simulation model, we restructure the spherical target and UCA system in Section 3. The observation area is filled with the seawater material whose conductivity is σ=4 S/m. Four spherical PEC electrodes are situated at (0.1,0,0), (0,0.1,0), (−0.1,0,0) and (0,−0.1,0), respectively, and one spherical PEC electrode is located at (0,0,0.0075) as the reference point. The radius of the five spherical PEC electrodes is 1 mm. Two electrodes with a radius of 1 mm are set at (0,0,−1.01) and (0,0,−0.99) as the emitters of the UCA system. The distortion of the field by the electrodes can be neglected because the volume is small enough. We load the source excitation of 50 A on the two electrodes, resulting in a dipole moment of 1 A·m. The spherical PEC target with a radius of 0.05 m is located in the observation area. The simulation model is shown in Figure 11.

**Figure 12:**
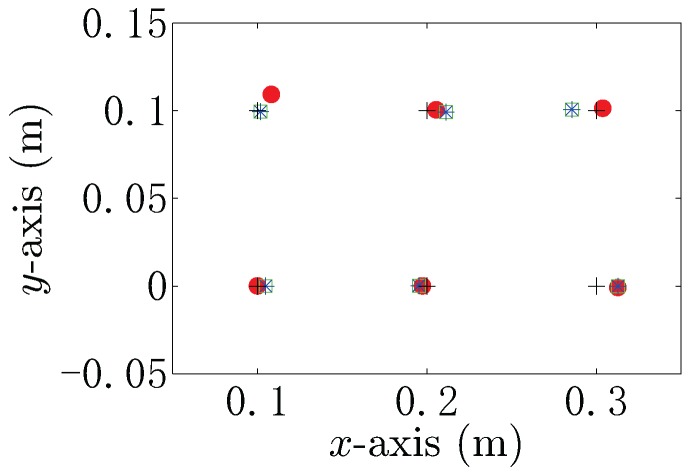
The estimated position by using the BEM model, simplified model and Rasnow model with the measured voltage data from the UCA system in the CST simulation by using the MP-MUSIC algorithm.

Figure 12 shows the accurate position and the estimated position of the target by using the BEM model, simplified model and Rasnow model with the measured voltage data from the UCA system. In Figure 12, the black crosses represent the actual position of the target, the red dots denote the estimated positions by using the BEM model, the green box represents the estimated positions by using the simplified model and the blue stars are the estimated positions by using the Rasnow model. It is easy to find that the simplified model and the Rasnow model provide the same locating performance. The comparisons between estimated positions and the actual positions are also given in Table 4. The estimation error by using the BEM model is 0.027 m, whereas the estimation error by using the simplified model is 0.015 m, which verify the conclusion that the simplified model and Rasnow model show better locating performance than that of the BEM model when 0.2≤x≤0.8. It should be noted that there are estimation errors in the simulation model. These may be the due to the following reasons. Firstly, the emitter and the receiving electrodes of the UCA system in the simulation model in CST are not ideal electrodes, which have physical dimensions in practice and could have some influence on the induction field. Secondly, the mesh of the simulation experiment area in CST would result in field calculation error, which can affect the voltage data measured by the UCA system. Compared with the Rasnow model, the estimated positions are in the acceptable region by using the proposed methods, and these results prove that the proposed localization scheme can be used as a precise metallic target locating sensors in underwater environments.

## 5. Experiment

**Figure 13:**
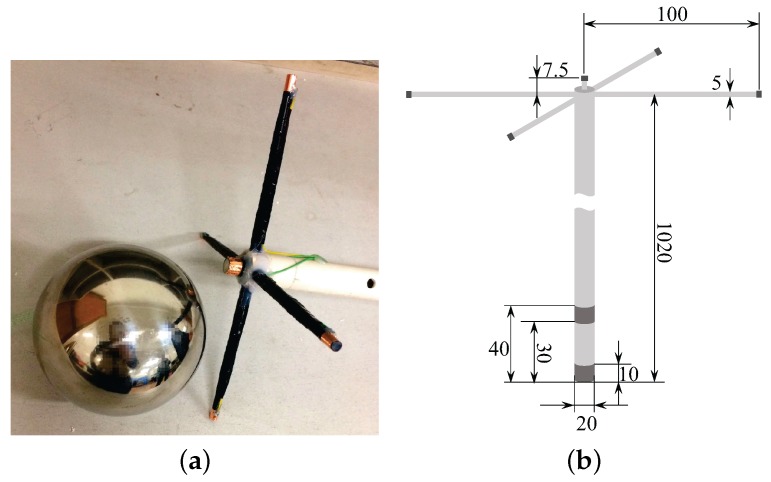
(**a**)The image of the UCA system with the conductor target; (**b**) the physical size of the UCA system (units: mm).

**Figure 14:**
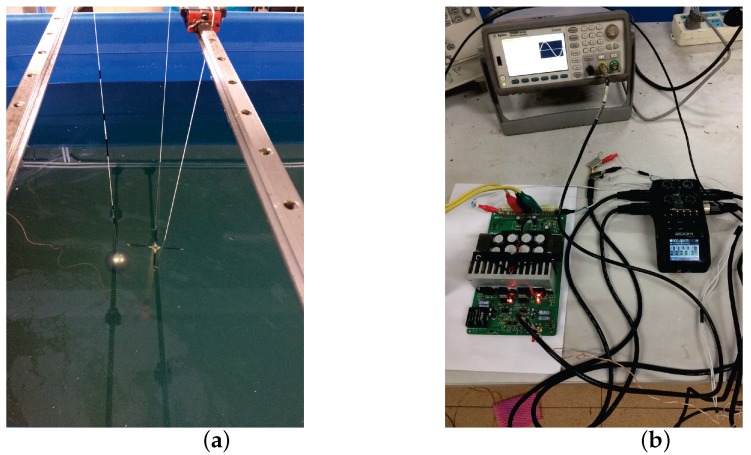
(**a**) The image of the experiment environment; (**b**) the amplifier of the electro-locator and the ZOOM H6 Handy Recorder.

In order to further verify the effectiveness of the proposed locating method in reality, a set of physical experiments is developed in our laboratory environment. We create a UCA system with 5 electrodes and an electric dipole source. The image of the UCA system and the detailed sizes are shown in Figure 13. A cylindrical plastic pipe is used as the frame of the UCA system, with a diameter of 20 mm. Two metal sheets cover the end of the cylindrical plastic pipe to act as the electrodes of the dipole source. Four plastic cylinders with diameters of 5 mm and lengths of 100 mm are fixed at the other end of the frame. The four plastic cylinders are set at the same plane, which is perpendicular to the frame. Four metal sheets cover the end of the plastic cylinders as the receiving electrodes. The fifth electrode or the reference electrode is situated at the top end of the frame, which is 7.5 mm from the plane of the four receiving electrodes. In Figure 14, the UCA system is vertically set at the center of the water tank, the electric dipole source of which is powered by a high power amplifier. The 5 electrodes compose the four-channel receiver, which are connected to a voltage measurement device product, the ZOOM H6 Handy Recorder. The measured data in the ZOOM H6 Handy Recorder will be imported into the MATLAB calculator. Then, the calculator outputs the estimated result of the target. The depth of the water in the tank is 1.5 m, and the conductivity of the water is set to be 4 S/m, which is close to the conductivity of sea water. A spherical target with a diameter of 100 mm is mounted on a horizontal movable gantry workbench with a size of 3 m by 1.5 m, which allows us to move the target along the pre-programmed trail with high geometric resolution.

**Figure 15:**
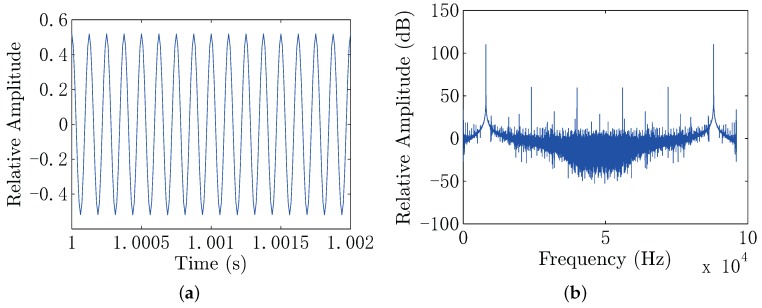
(**a**) The measured voltage in the time domain; (**b**) the frequency spectrum of the measured voltage.

**Figure 16:**
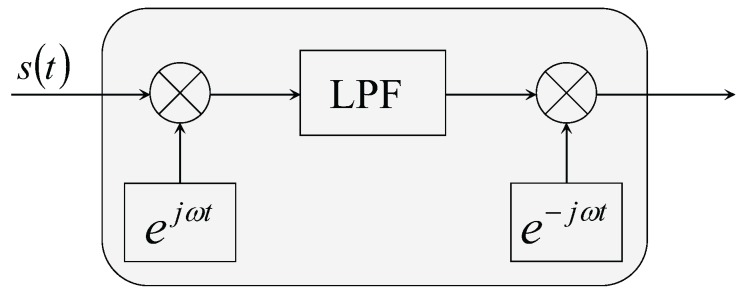
The schematic diagram of the canonical high Q bandpass filter.

**Figure 17:**
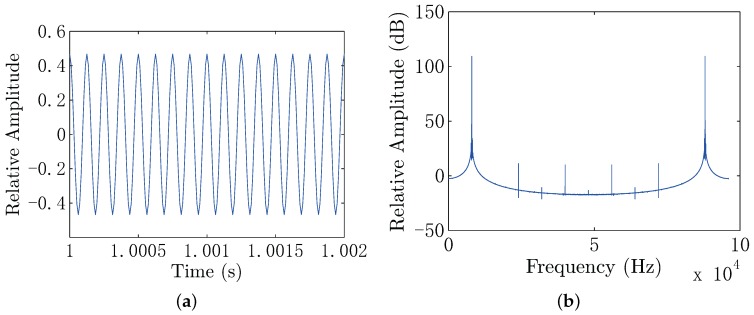
(**a**) The measured data after the high Q bandpass filter in the time domain; (**b**) the frequency spectrum of the measured data after the filter.

In this experiment, the frequency of the electric dipole source is 8 kHz, and the impedance of the electric dipole source is 6.53Ω. The output voltage of the power stage is 15.5 Vrms, yielding the output power of 36.8 W and dipole moment of 0.1 A·m. The positions of the electrodes in the UCA system are known and fixed, which are listed in Table 1. During the measurement, we first set the conductor target at the point (x,0,0.1), where x=0.1,0.2,0.3and0.4. As the voltage of each channel is weak in seawater, we use the ZOOM H6 Handy Recorder as the analog to digital converter (ADC) device, which has configurable gain from −∞–55.5 dB with a 96-kHz sample rate and 24-bit precision. We also do the same process for the insulator target. In this experiment, the measured voltage range of each channel is within 0.1∼15.6 mV. The measured signal contains noise shown in Figure 15, where the noise in the sea water is low, because of the high conductivity of the sea water, significantly shielding the electric noise and interference. In order to further filter the noise and interference, the digital filter is used in the background. The parameters of the digital filter of each channel are the same, which will not introduce additional gain distortion compared with the hardware filter on the front end. In this electro-locator system, the canonical high Q bandpass filter is used. We first shift the signal to the baseband. Then, we filter the baseband signal with a narrow band low pass filter, for which the coefficients are designed by the use of MATLAB FDATOOL. After that, the signal is shifted to the original frequency point. The canonical high Q bandpass filter is shown in Figure 16, with the bandpass of 400 Hz. The filtered data are shown in Figure 17, where the noise and interference are reduced after the digital filter.

**Figure 18:**
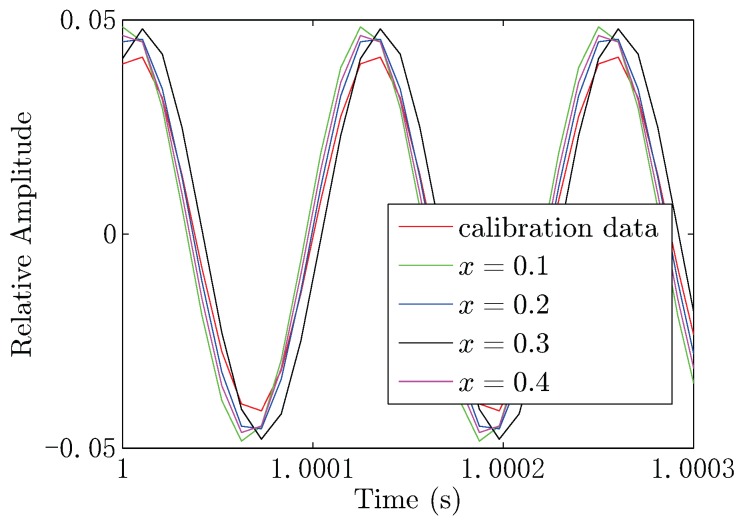
The calibration data and the measured voltages when the target is situated at points (x,0,0.1) for the first receiving channel.

In the practical situation, there will be position error when building the UCA system. To overcome this drawback, we add calibration during the locating process. The calibration data are obtained without the target in the sea water tank. Then, we measure the voltages when the target is situated in the tank shown in Figure 18. It can be seen from Figure 18 that the voltages vary when the target is placed at different positions in the tank. During the locating process, the input data are the difference between the measured data and the calibration data. Here, we give an example of the location process according to one part of the raw data, and the electro-locator will finally output the target position result.

**Figure 19:**
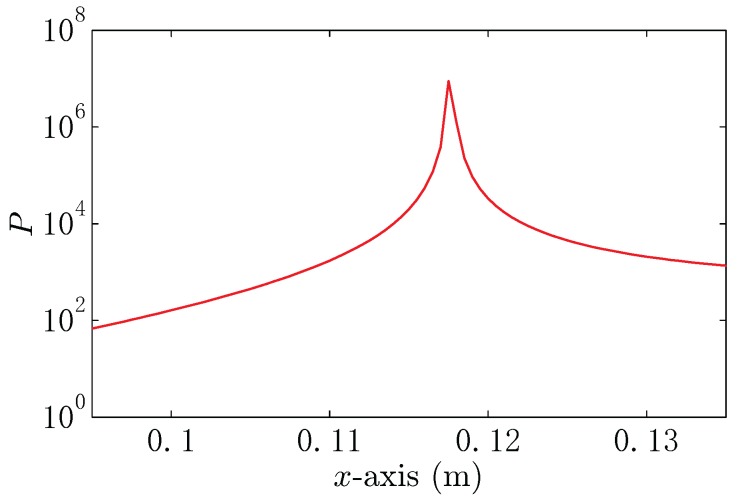
The space spectrum of the electro-locator at the hypothesis point (x,0,0.07).

- Step 1: Load the boundary matrix $Z_{s}^{s}$. Load the measured data $\Phi_{m}$ and the calibration data $\Phi_{r}$ from the folders “./newx1CHn_ins” and “./newCHn_ref_ins”, which can be downloaded from https://drive.google.com/open?id=15hPIKZaSmfmfFeUPHwut9Lb2EXugKXZF;
- Step 2: Calculate the input data, the matrix $\Phi \left(t\right)=\Phi_{m}-\Phi_{r}$, yielding the covariance matrix $R_{\Phi }=\alpha \left(\begin{array}{cccc} 0.1862 & 0.8761 & 1.0896 & 0.6338\\ 0.8761 & 4.1213 & 5.1256 & 2.9814\\ 1.0896 & 5.1256 & 6.3746 & 3.7079\\ 0.6338 & 2.9814 & 3.7079 & 2.1568 \end{array}\right)$, where α is a factor lager than zero;
- Step 3: Get the eigenvector U and eigenvalue Σ by eigenvalue-decomposition. Here, we have $U=\left(\begin{array}{cccc} -0.0891 & 0.6867 & 0.7114 & 0.1204\\ -0.1714 & -0.6354 & 0.4959 & 0.5666\\ -0.3745 & 0.3420 & -0.4963 & 0.7046\\ 0.9069 & 0.0886 & -0.0413 & 0.4099 \end{array}\right)$, $\Sigma =\beta \left(\begin{array}{cccc} 0 & 0 & 0 & 0\\ 0 & 0 & 0 & 0\\ 0 & 0 & 0 & 0\\ 0 & 0 & 0 & 1.2839 \end{array}\right)$, where β is a factor lager than zero. Thus, the signal subspace is $U_{S}=\left(\begin{array}{c} 0.1204\\ 0.5666\\ 0.7046\\ 0.4099 \end{array}\right)$, and the noise subspace projection matrix is $P^{⊥}=\left(\begin{array}{cccc} 0.9855 & -0.0682 & -0.0849 & -0.0494\\ -0.0682 & 0.6790 & -0.3992 & -0.2322\\ -0.0849 & -0.3992 & 0.5035 & -0.2888\\ -0.0494 & -0.2322 & -0.2888 & 0.8320 \end{array}\right)$;
- Step 4: Scan the observation area where the target exists with a series locating hypothesis and calculate the space spectrum. Here, we give the space spectrum near the target, which is shown in Figure 19;
- Step 5: From Figure 19, we can see that the position corresponding to the peak of the spectrum is (0.117,0,0.07), which is close to the true position (0.1,0,0.1).

The estimated positions and their actual positions are also given in Table 5, and we can find that the maximum location error is 0.055 m for the conductor target and 0.069 m for the insulator target. However, the location errors of the other points are less than 0.05 m. The estimation errors may be due to the following reasons. Firstly, the electric dipole source in the UCA system is not an ideal dipole, which has physical dimension errors in practice. Secondly, the frame of the UCA system can affect the distribution of the electric field. Thirdly, the electrodes’ position deviations are introduced to build the UCA system, resulting in locating errors. Finally, the actual positions of the target and the UCA system may be slightly moved during measurement because of the water wave. Although some experimental results slightly offset the center position, the localization accuracy is still good. These results prove that the proposed localization scheme can be applied to underwater target locating.

## 6. Conclusions

In this paper, we propose a novel target locating method based on the MP-MUSIC algorithm in the underwater environment. The BEM is introduced to discretize the continuous Green’s function and transpose it to a matrix. By solving the matrix, the induction field of the target is accurately described. However, locating the target by using the proposed BEM model requires prior information of the size of the target, which may restrict the use of the locating method. To overcome this drawback, we propose a simplified model, in which the prior information of the size of the target is not needed. According to the comparison with the canonical MUSIC algorithm, we find that the MP-MUSIC can provide better locating performance. A UCA system is also proposed, on which the receiving electrodes are all situated at the equipotential points. The specially-designed UCA system provides the feature that the receiving electrodes cannot sense the voltage from the emitter of the UCA system, but could sense the voltage from the target. A numerical example and the corresponding simulation model via CST are carried out. The noise, the distance between the target and the UCA system and the number of receiving electrodes of the UCA system were investigated to analyze the effect on the locating performance in detail by using the proposed models. According to the comparison, we found that the simplified model has the same locating performance as the Rasnow model by using the MP-MUSIC. The simulation results showed that the simplified model can provide better locating performance when 0.2≤x≤0.8, and the BEM model can give better locating performance when x≤0.2 and x≥0.8. However, both models provide unsatisfactory locating performance when the target gets close to the center of the UCA. The numerical results also show that the locating performance is not sensitive to the azimuth of the target when using the locating method based on the BEM with the MP-MUSIC algorithm. In addition, the optimal number of electrodes on circle *l* of the UCA system is four, which gives the best locating performance. A set of physical experiments is carried out for locating the conductor and insulator target. The results of the experiment verified the effectiveness of the proposed locating method. In our further work, we will deign a new UCA system and improve the locating method to acquire much more accurate results.

## Figures and Tables

**Table 1:** The positions of the five electrodes in the UCA system.

Index	1	2	3	4	5
*x* (m)	0.1	0.0	−0.1	0.0	0.0
*y* (m)	0.0	0.1	0.0	−0.1	0.0
*z* (m)	0.0	0.0	0.0	0.0	0.0075

**Table 2:** The RMS errors of the position estimated versus different SNRs.

*x*-Axis (m)	0	0.1	0.2	0.3	0.8	1.3
BEM model, SNR=15 dB	0.238	0	0.006	0.012	0.019	0.011
BEM model, SNR=20 dB	0.122	0	0.005	0.007	0.012	0.007
BEM model, SNR=25 dB	0.081	0	0.003	0.005	0.008	0.004
BEM model, SNR=30 dB	0.051	0	0.003	0.004	0.004	0.003
simplified model, SNR=15 dB	0.235	0.004	0.009	0.008	0.025	0.137
simplified model, SNR=20 dB	0.187	0.003	0.005	0.004	0.019	0.136
simplified model, SNR=25 dB	0.201	0.003	0.004	0.002	0.016	0.130
simplified model, SNR=30 dB	0.209	0.003	0.003	0.002	0.015	0.133

**Table 3:** The locating errors when the azimuths are 0 degrees and 45 degrees.

*r* (m)		0.1	0.2	0.3	0.7	0.8	1.0
BEM model, MP-MUSIC	0 deg	0	0	0	0.011	0.012	0.017
	45 deg	0	0.001	0.003	0.005	0.008	0.029
Rasnow model, MP-MUSIC	0 deg	0.004	0.002	0.001	0.010	0.018	0.065
	45 deg	0.002	0.002	0.002	0.010	0.019	0.065
BEM model, canonical MUSIC	0 deg	0	0	0	0.015	0.044	0.127
	45 deg	0.001	0.004	0.022	0.377	0.464	0.674
Rasnow model, canonical MUSIC	0 deg	0.002	0.003	0.007	0.270	0.328	0.456
	45 deg	0.066	0.127	0.156	0.279	0.397	0.733

**Table 4:** The estimated positions of the electric dipole source in different positions.

Model	Actual Position	Estimated Position	Error (m)
BEM model	(0.1,0,0.1)	(0.100,0,0.100)	0
	(0.2,0,0.1)	(0.197,0,0.102)	0.004
	(0.3,0,0.1)	(0.313,−0.001,0.124)	0.027
	(0.1,0.1,0.1)	(0.108,0.109,0.082)	0.022
	(0.2,0.1,0.1)	(0.206,0.101,0.100)	0.006
	(0.3,0.1,0.1)	(0.304,0.101,0.100)	0.004
Simplified model	(0.1,0,0.1)	(0.105,0,0.100)	0.005
	(0.2,0,0.1)	(0.195,0,0.101)	0.005
	(0.3,0,0.1)	(0.313,0,0.102)	0.013
	(0.1,0.1,0.1)	(0.102,0.1,0.102)	0.003
	(0.2,0.1,0.1)	(0.211,0.099,0.102)	0.012
	(0.3,0.1,0.1)	(0.286,0.101,0.104)	0.015
Rasnow model	(0.1,0,0.1)	(0.105,0,0.100)	0.005
	(0.2,0,0.1)	(0.195,0,0.101)	0.005
	(0.3,0,0.1)	(0.313,0,0.102)	0.013
	(0.1,0.1,0.1)	(0.102,0.1,0.102)	0.003
	(0.2,0.1,0.1)	(0.211,0.099,0.102)	0.012
	(0.3,0.1,0.1)	(0.286,0.101,0.104)	0.015

**Table 5:** The estimated positions of the conductor target and insulator target in different positions.

Target	Actual Position	Estimated Position	Error (m)
conductor	(0.1,0,0.1)	(0.053,0,0.129)	0.055
	(0.2,0,0.1)	(0.181,−0.01,0.103)	0.022
	(0.3,0,0.1)	(0.324,0.01,0.106)	0.027
	(0.4,0,0.1)	(0.373,0.01,0.102)	0.029
insulator	(0.1,0,0.1)	(0.087,0,0.110)	0.016
	(0.2,0,0.1)	(0.220,−0.001,0.105)	0.021
	(0.3,0,0.1)	(0.252,0.001,0.104)	0.048
	(0.4,0,0.1)	(0.331,0,0.104)	0.069
